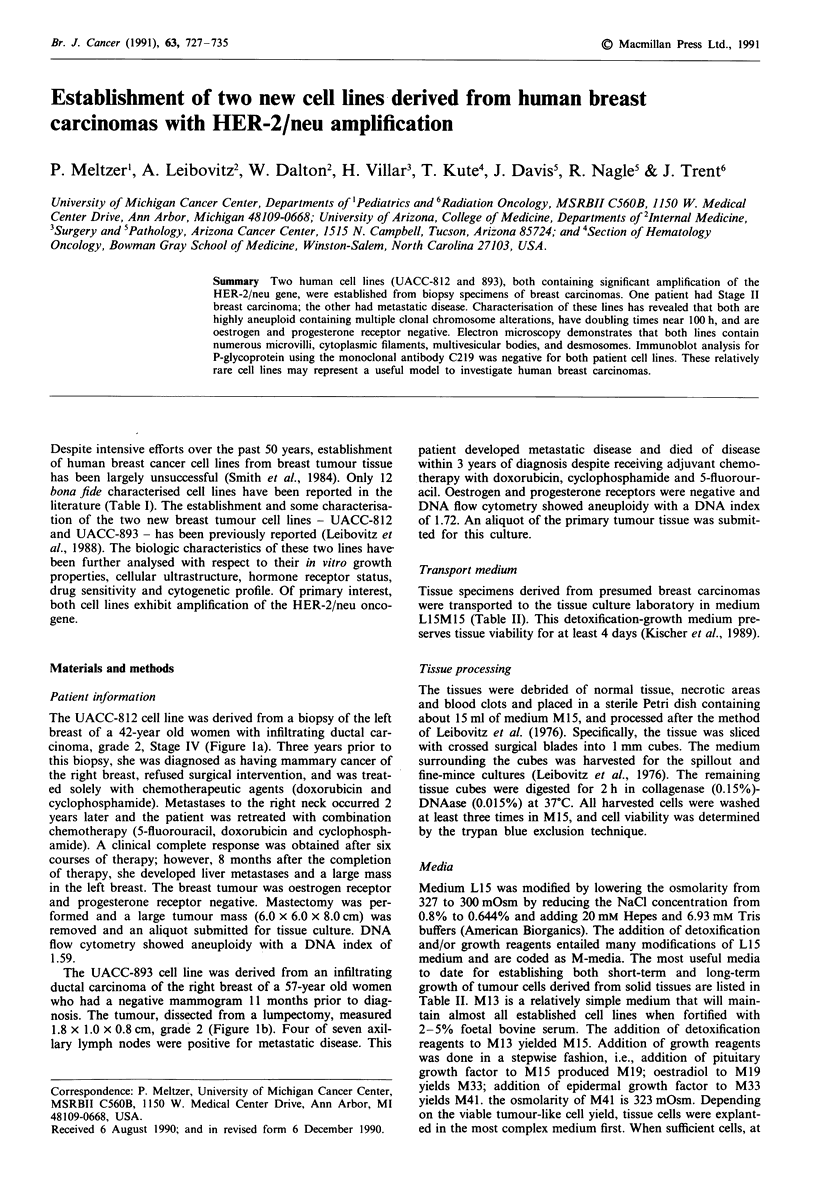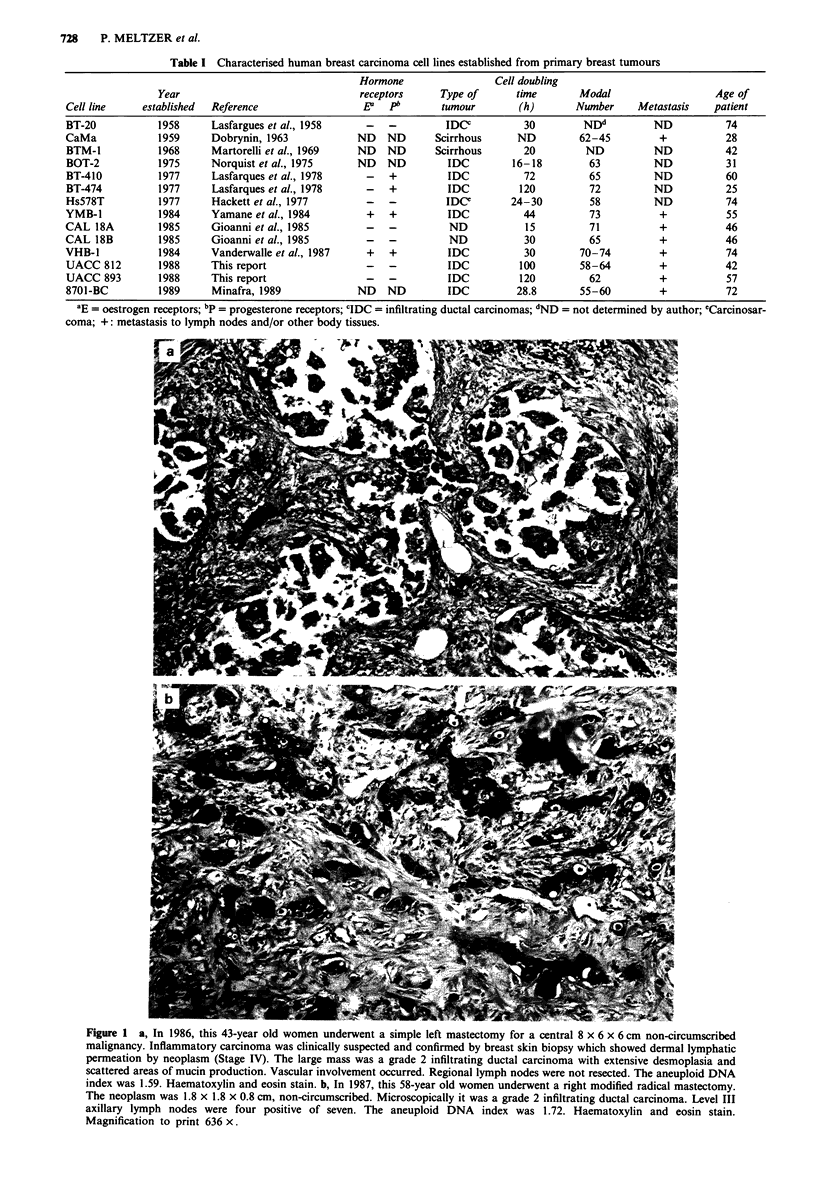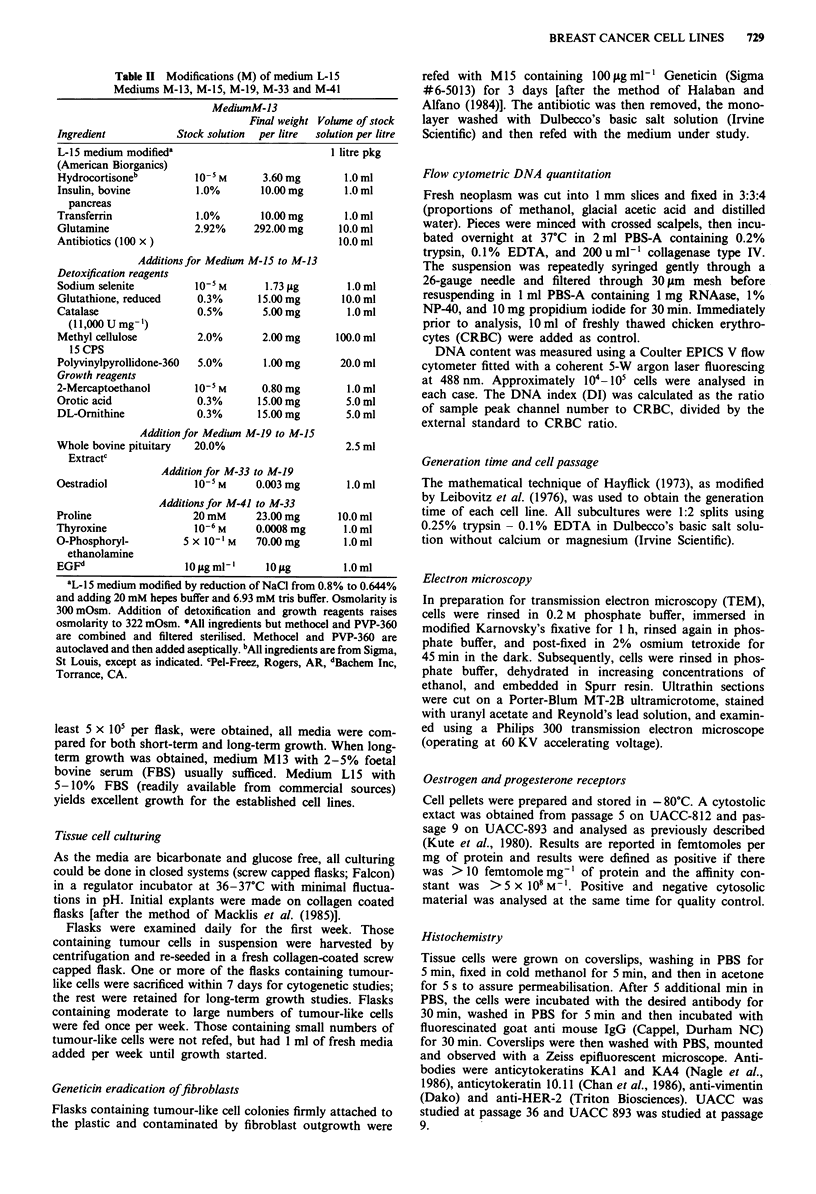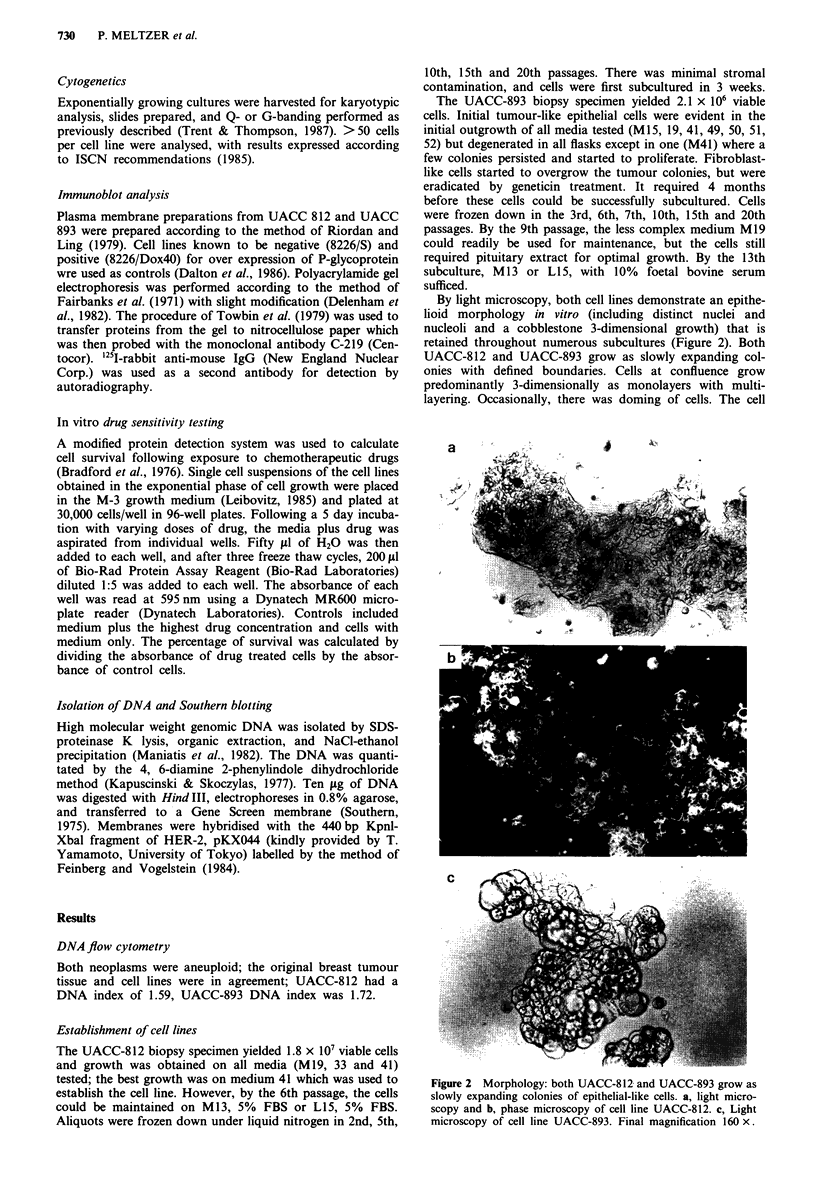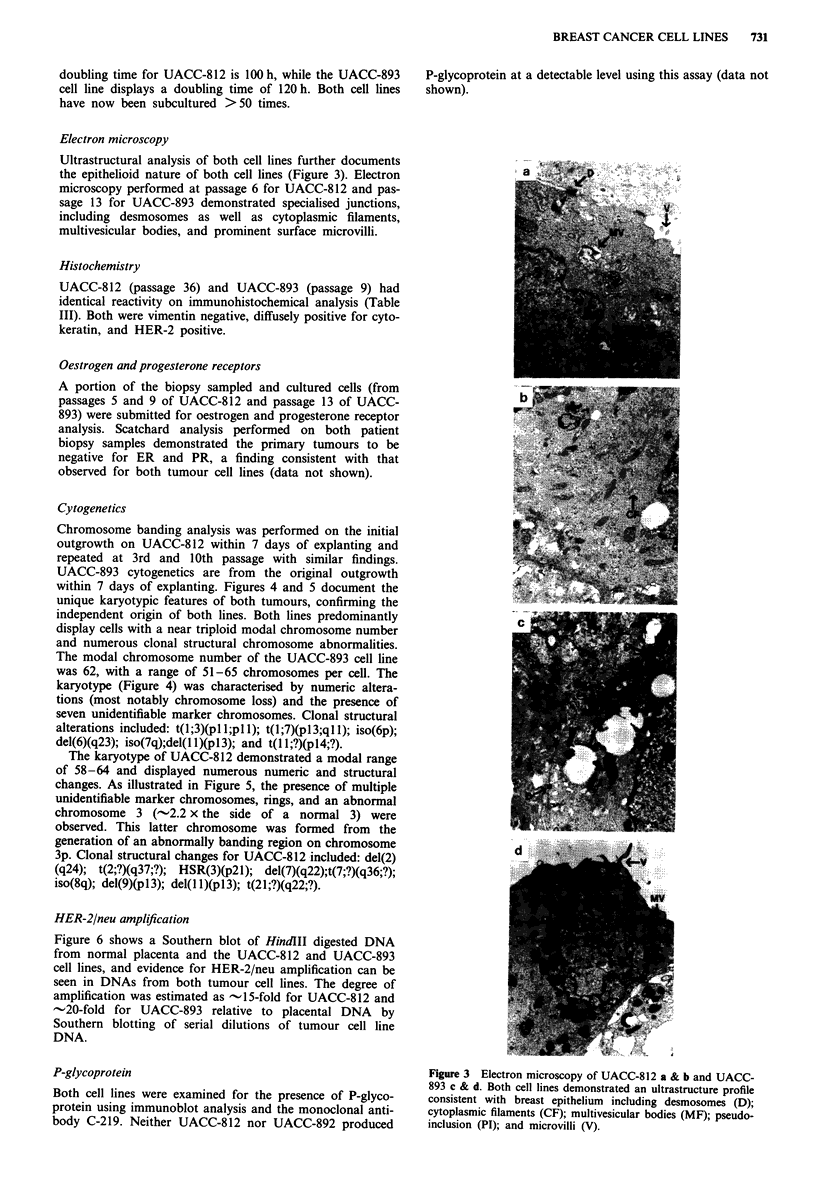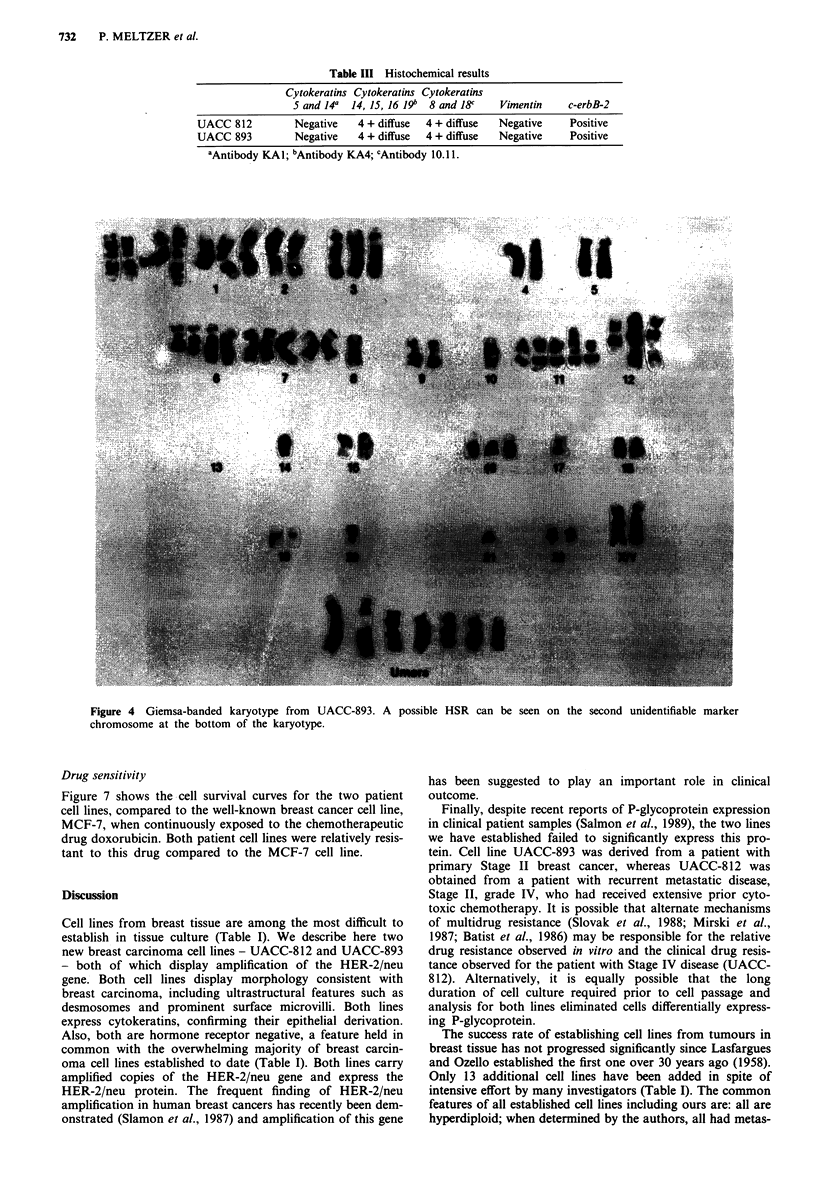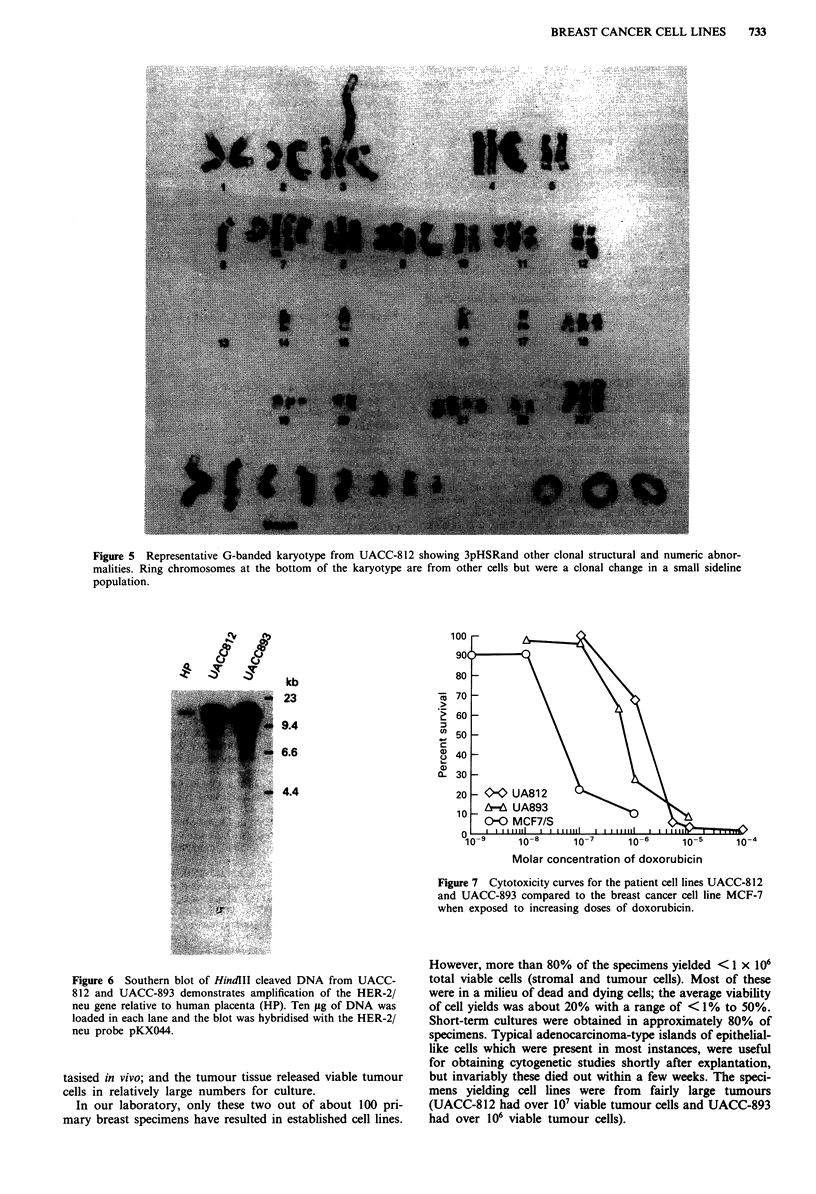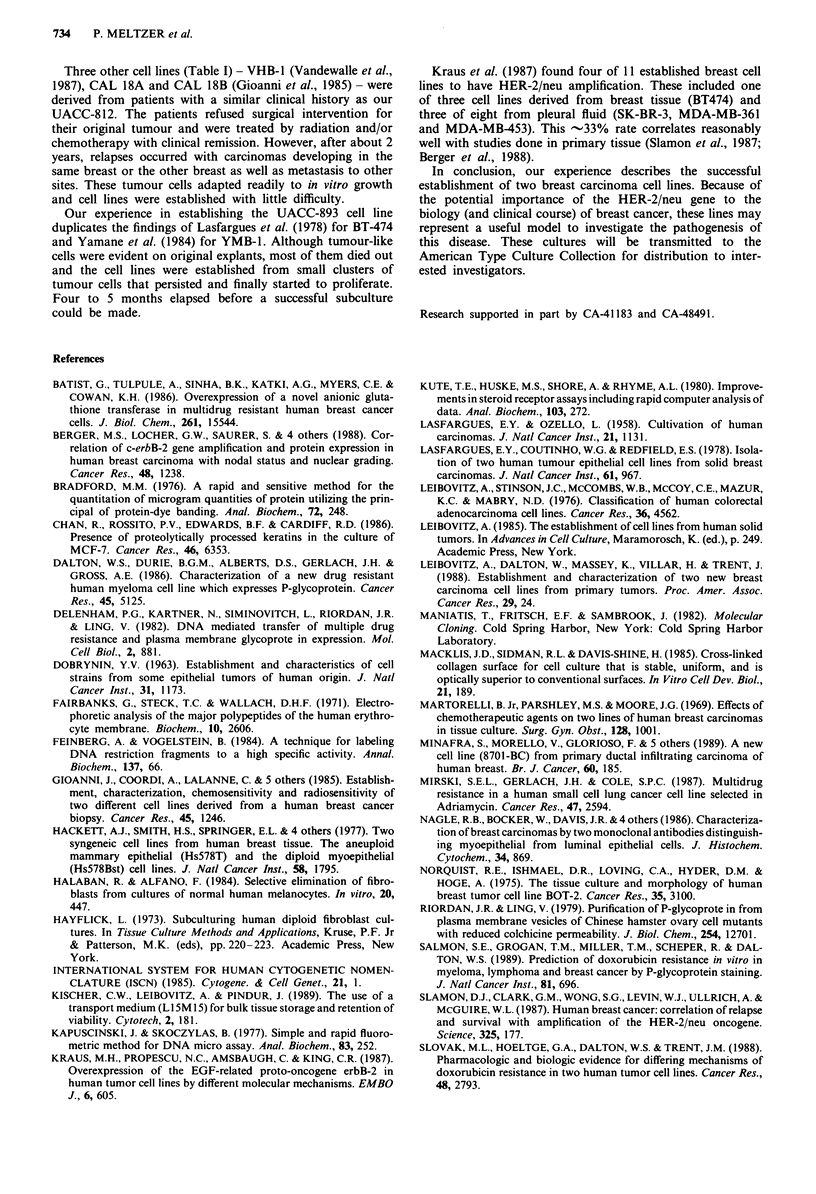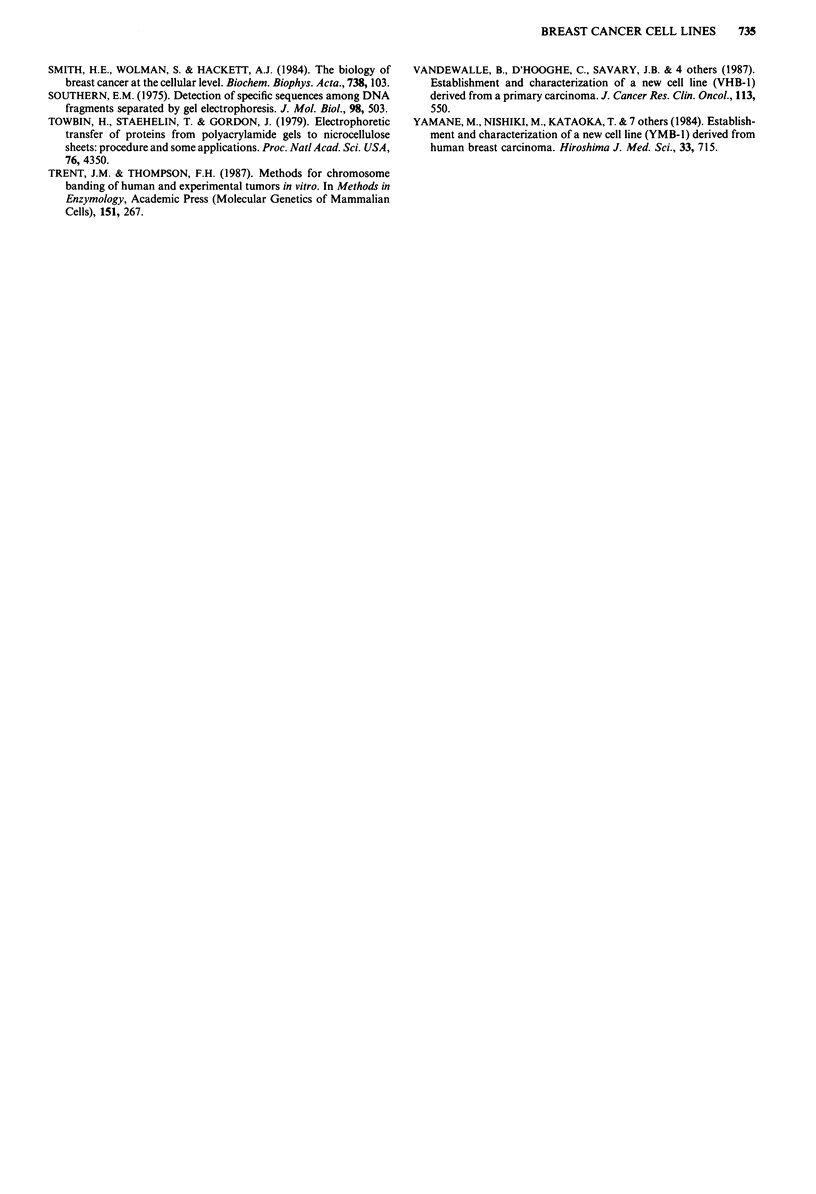# Establishment of two new cell lines derived from human breast carcinomas with HER-2/neu amplification.

**DOI:** 10.1038/bjc.1991.164

**Published:** 1991-05

**Authors:** P. Meltzer, A. Leibovitz, W. Dalton, H. Villar, T. Kute, J. Davis, R. Nagle, J. Trent

**Affiliations:** University of Michigan Cancer Center, Department of Pediatrics, Ann Arbor 48109-0668.

## Abstract

**Images:**


Br. J. Cancer (1991), 63, 727–735 © Macmillan Press Ltd., 1991

### Establishment of two new cell lines derived from human breast carcinomas with HER-2/neu amplification

P. Meltzer[1], A. Leibovitz[2], W. Dalton[2], H. Villar[3], T. Kute[4], J. Davis[5], R. Nagle[5] & J. Trent[6]

*University of Michigan Cancer Center, Departments of [1]Pediatrics and [6]Radiation Oncology, MSRBII C560B, 1150 W. Medical Center Drive, Ann Arbor, Michigan 48109-0668; University of Arizona, College of Medicine, Departments of [2]Internal Medicine, [3]Surgery and [5]Pathology, Arizona Cancer Center, 1515 N. Campbell, Tucson, Arizona 85724; and [4]Section of Hematology Oncology, Bowman Gray School of Medicine, Winston-Salem, North Carolina 27103, USA.*

**Summary** Two human cell lines (UACC-812 and 893), both containing significant amplification of the HER-2/neu gene, were established from biopsy specimens of breast carcinomas. One patient had Stage II breast carcinoma; the other had metastatic disease. Characterisation of these lines has revealed that both are highly aneuploid containing multiple clonal chromosome alterations, have doubling times near 100 h, and are oestrogen and progesterone receptor negative. Electron microscopy demonstrates that both lines contain numerous microvilli, cytoplasmic filaments, multivesicular bodies, and desmosomes. Immunoblot analysis for P-glycoprotein using the monoclonal antibody C219 was negative for both patient cell lines. These relatively rare cell lines may represent a useful model to investigate human breast carcinomas.

Despite intensive efforts over the past 50 years, establishment of human breast cancer cell lines from breast tumour tissue has been largely unsuccessful (Smith *et al.*, 1984). Only 12 *bona fide* characterised cell lines have been reported in the literature (Table I). The establishment and some characterisation of the two new breast tumour cell lines – UACC-812 and UACC-893 – has been previously reported (Leibovitz *et al.*, 1988). The biologic characteristics of these two lines have been further analysed with respect to their *in vitro* growth properties, cellular ultrastructure, hormone receptor status, drug sensitivity and cytogenetic profile. Of primary interest, both cell lines exhibit amplification of the HER-2/neu oncogene.

### Materials and methods

### *Patient information*

The UACC-812 cell line was derived from a biopsy of the left breast of a 42-year old women with infiltrating ductal carcinoma, grade 2, Stage IV (Figure 1a). Three years prior to this biopsy, she was diagnosed as having mammary cancer of the right breast, refused surgical intervention, and was treated solely with chemotherapeutic agents (doxorubicin and cyclophosphamide). Metastases to the right neck occurred 2 years later and the patient was retreated with combination chemotherapy (5-fluorouracil, doxorubicin and cyclophosphamide). A clinical complete response was obtained after six courses of therapy; however, 8 months after the completion of therapy, she developed liver metastases and a large mass in the left breast. The breast tumour was oestrogen receptor and progesterone receptor negative. Mastectomy was performed and a large tumour mass (6.0 × 6.0 × 8.0 cm) was removed and an aliquot submitted for tissue culture. DNA flow cytometry showed aneuploidy with a DNA index of 1.59.

The UACC-893 cell line was derived from an infiltrating ductal carcinoma of the right breast of a 57-year old women who had a negative mammogram 11 months prior to diagnosis. The tumour, dissected from a lumpectomy, measured 1.8 × 1.0 × 0.8 cm, grade 2 (Figure 1b). Four of seven axillary lymph nodes were positive for metastatic disease. This patient developed metastatic disease and died of disease within 3 years of diagnosis despite receiving adjuvant chemotherapy with doxorubicin, cyclophosphamide and 5-fluorouracil. Oestrogen and progesterone receptors were negative and DNA flow cytometry showed aneuploidy with a DNA index of 1.72. An aliquot of the primary tumour tissue was submitted for this culture.

### *Transport medium*

Tissue specimens derived from presumed breast carcinomas were transported to the tissue culture laboratory in medium L15M15 (Table II). This detoxification-growth medium preserves tissue viability for at least 4 days (Kischer *et al.*, 1989).

### *Tissue processing*

The tissues were debrided of normal tissue, necrotic areas and blood clots and placed in a sterile Petri dish containing about 15 ml of medium M15, and processed after the method of Leibovitz *et al.* (1976). Specifically, the tissue was sliced with crossed surgical blades into 1 mm cubes. The medium surrounding the cubes was harvested for the spillout and fine-mince cultures (Leibovitz *et al.*, 1976). The remaining tissue cubes were digested for 2 h in collagenase (0.15%)-DNAase (0.015%) at 37°C. All harvested cells were washed at least three times in M15, and cell viability was determined by the trypan blue exclusion technique.

### *Media*

Medium L15 was modified by lowering the osmolarity from 327 to 300 mOsm by reducing the NaCl concentration from 0.8% to 0.644% and adding 20 mM Hepes and 6.93 mM Tris buffers (American Biorganics). The addition of detoxification and/or growth reagents entailed many modifications of L15 medium and are coded as M-media. The most useful media to date for establishing both short-term and long-term growth of tumour cells derived from solid tissues are listed in Table II. M13 is a relatively simple medium that will maintain almost all established cell lines when fortified with 2–5% foetal bovine serum. The addition of detoxification reagents to M13 yielded M15. Addition of growth reagents was done in a stepwise fashion, i.e., addition of pituitary growth factor to M15 produced M19; oestradiol to M19 yields M33; addition of epidermal growth factor to M33 yields M41. the osmolarity of M41 is 323 mOsm. Depending on the viable tumour-like cell yield, tissue cells were explanted in the most complex medium first. When sufficient cells, at

Correspondence: P. Meltzer, University of Michigan Cancer Center, MSRBII C560B, 1150 W. Medical Center Drive, Ann Arbor, MI 48109-0668, USA.
Received 6 August 1990; and in revised form 6 December 1990.

**Table I** Characterised human breast carcinoma cell lines established from primary breast tumours

| Cell line | Year established | Reference | Hormone receptors $E^a$ | $P^b$ | Type of tumour | Cell doubling time (h) | Modal Number | Metastasis | Age of patient |
|---|---|---|---|---|---|---|---|---|---|
| BT-20 | 1958 | Lasfargues *et al.*, 1958 | – | – | IDC[c] | 30 | ND[d] | ND | 74 |
| CaMa | 1959 | Dobrynin, 1963 | ND | ND | Scirrhous | ND | 62–45 | + | 28 |
| BTM-1 | 1968 | Martorelli *et al.*, 1969 | ND | ND | Scirrhous | 20 | ND | ND | 42 |
| BOT-2 | 1975 | Norquist *et al.*, 1975 | ND | ND | IDC | 16–18 | 63 | ND | 31 |
| BT-410 | 1977 | Lasfarques *et al.*, 1978 | – | + | IDC | 72 | 65 | ND | 60 |
| BT-474 | 1977 | Lasfarques *et al.*, 1978 | – | + | IDC | 120 | 72 | ND | 25 |
| Hs578T | 1977 | Hackett *et al.*, 1977 | – | – | IDC[e] | 24–30 | 58 | ND | 74 |
| YMB-1 | 1984 | Yamane *et al.*, 1984 | + | + | IDC | 44 | 73 | + | 55 |
| CAL 18A | 1985 | Gioanni *et al.*, 1985 | – | – | ND | 15 | 71 | + | 46 |
| CAL 18B | 1985 | Gioanni *et al.*, 1985 | – | – | ND | 30 | 65 | + | 46 |
| VHB-1 | 1984 | Vanderwalle *et al.*, 1987 | + | + | IDC | 30 | 70–74 | + | 74 |
| UACC 812 | 1988 | This report | – | – | IDC | 100 | 58–64 | + | 42 |
| UACC 893 | 1988 | This report | – | – | IDC | 120 | 62 | + | 57 |
| 8701-BC | 1989 | Minafra, 1989 | ND | ND | IDC | 28.8 | 55–60 | + | 72 |

[a]E = oestrogen receptors; [b]P = progesterone receptors; [c]IDC = infiltrating ductal carcinomas; [d]ND = not determined by author; [e]Carcinosarcoma; +: metastasis to lymph nodes and/or other body tissues.

**Figure 1** **a**, In 1986, this 43-year old women underwent a simple left mastectomy for a central 8 × 6 × 6 cm non-circumscribed malignancy. Inflammatory carcinoma was clinically suspected and confirmed by breast skin biopsy which showed dermal lymphatic permeation by neoplasm (Stage IV). The large mass was a grade 2 infiltrating ductal carcinoma with extensive desmoplasia and scattered areas of mucin production. Vascular involvement occurred. Regional lymph nodes were not resected. The aneuploid DNA index was 1.59. Haematoxylin and eosin stain. **b**, In 1987, this 58-year old women underwent a right modified radical mastectomy. The neoplasm was 1.8 × 1.8 × 0.8 cm, non-circumscribed. Microscopically it was a grade 2 infiltrating ductal carcinoma. Level III axillary lymph nodes were four positive of seven. The aneuploid DNA index was 1.72. Haematoxylin and eosin stain. Magnification to print 636 ×.

**Table II** Modifications (M) of medium L-15
Mediums M-13, M-15, M-19, M-33 and M-41

| Ingredient | Stock solution | Final weight per litre | Volume of stock solution per litre |
|---|---|---|---|
| *Medium M-13* | | | |
| L-15 medium modified[a] (American Biorganics) | | | 1 litre pkg |
| Hydrocortisone[b] | $10^{-5}$ M | 3.60 mg | 1.0 ml |
| Insulin, bovine pancreas | 1.0% | 10.00 mg | 1.0 ml |
| Transferrin | 1.0% | 10.00 mg | 1.0 ml |
| Glutamine | 2.92% | 292.00 mg | 10.0 ml |
| Antibiotics (100 × ) | | | 10.0 ml |
| *Additions for Medium M-15 to M-13* | | | |
| *Detoxification reagents* | | | |
| Sodium selenite | $10^{-5}$ M | 1.73 µg | 1.0 ml |
| Glutathione, reduced | 0.3% | 15.00 mg | 10.0 ml |
| Catalase (11,000 U $mg^{-1}$) | 0.5% | 5.00 mg | 1.0 ml |
| Methyl cellulose 15 CPS | 2.0% | 2.00 mg | 100.0 ml |
| Polyvinylpyrollidone-360 | 5.0% | 1.00 mg | 20.0 ml |
| *Growth reagents* | | | |
| 2-Mercaptoethanol | $10^{-5}$ M | 0.80 mg | 1.0 ml |
| Orotic acid | 0.3% | 15.00 mg | 5.0 ml |
| DL-Ornithine | 0.3% | 15.00 mg | 5.0 ml |
| *Addition for Medium M-19 to M-15* | | | |
| Whole bovine pituitary Extract[c] | 20.0% | | 2.5 ml |
| *Addition for M-33 to M-19* | | | |
| Oestradiol | $10^{-5}$ M | 0.003 mg | 1.0 ml |
| *Additions for M-41 to M-33* | | | |
| Proline | 20 mM | 23.00 mg | 10.0 ml |
| Thyroxine | $10^{-6}$ M | 0.0008 mg | 1.0 ml |
| O-Phosphoryl-ethanolamine | $5 \times 10^{-1}$ M | 70.00 mg | 1.0 ml |
| EGF[d] | 10 µg $ml^{-1}$ | 10 µg | 1.0 ml |

[a]L-15 medium modified by reduction of NaCl from 0.8% to 0.644% and adding 20 mM hepes buffer and 6.93 mM tris buffer. Osmolarity is 300 mOsm. Addition of detoxification and growth reagents raises osmolarity to 322 mOsm. *All ingredients but methocel and PVP-360 are combined and filtered sterilised. Methocel and PVP-360 are autoclaved and then added aseptically. [b]All ingredients are from Sigma, St Louis, except as indicated. [c]Pel-Freez, Rogers, AR, [d]Bachem Inc, Torrance, CA.

least $5 \times 10^5$ per flask, were obtained, all media were compared for both short-term and long-term growth. When long-term growth was obtained, medium M13 with 2–5% foetal bovine serum (FBS) usually sufficed. Medium L15 with 5–10% FBS (readily available from commercial sources) yields excellent growth for the established cell lines.

*Tissue cell culturing*

As the media are bicarbonate and glucose free, all culturing could be done in closed systems (screw capped flasks; Falcon) in a regulator incubator at 36–37°C with minimal fluctuations in pH. Initial explants were made on collagen coated flasks [after the method of Macklis *et al.* (1985)].

Flasks were examined daily for the first week. Those containing tumour cells in suspension were harvested by centrifugation and re-seeded in a fresh collagen-coated screw capped flask. One or more of the flasks containing tumour-like cells were sacrificed within 7 days for cytogenetic studies; the rest were retained for long-term growth studies. Flasks containing moderate to large numbers of tumour-like cells were fed once per week. Those containing small numbers of tumour-like cells were not refed, but had 1 ml of fresh media added per week until growth started.

*Geneticin eradication of fibroblasts*

Flasks containing tumour-like cell colonies firmly attached to the plastic and contaminated by fibroblast outgrowth were refed with M15 containing 100 µg $ml^{-1}$ Geneticin (Sigma #6-5013) for 3 days [after the method of Halaban and Alfano (1984)]. The antibiotic was then removed, the monolayer washed with Dulbecco's basic salt solution (Irvine Scientific) and then refed with the medium under study.

*Flow cytometric DNA quantitation*

Fresh neoplasm was cut into 1 mm slices and fixed in 3:3:4 (proportions of methanol, glacial acetic acid and distilled water). Pieces were minced with crossed scalpels, then incubated overnight at 37°C in 2 ml PBS-A containing 0.2% trypsin, 0.1% EDTA, and 200 u $ml^{-1}$ collagenase type IV. The suspension was repeatedly syringed gently through a 26-gauge needle and filtered through 30 µm mesh before resuspending in 1 ml PBS-A containing 1 mg RNAase, 1% NP-40, and 10 mg propidium iodide for 30 min. Immediately prior to analysis, 10 ml of freshly thawed chicken erythrocytes (CRBC) were added as control.

DNA content was measured using a Coulter EPICS V flow cytometer fitted with a coherent 5-W argon laser fluorescing at 488 nm. Approximately $10^4$–$10^5$ cells were analysed in each case. The DNA index (DI) was calculated as the ratio of sample peak channel number to CRBC, divided by the external standard to CRBC ratio.

*Generation time and cell passage*

The mathematical technique of Hayflick (1973), as modified by Leibovitz *et al.* (1976), was used to obtain the generation time of each cell line. All subcultures were 1:2 splits using 0.25% trypsin – 0.1% EDTA in Dulbecco's basic salt solution without calcium or magnesium (Irvine Scientific).

*Electron microscopy*

In preparation for transmission electron microscopy (TEM), cells were rinsed in 0.2 M phosphate buffer, immersed in modified Karnovsky's fixative for 1 h, rinsed again in phosphate buffer, and post-fixed in 2% osmium tetroxide for 45 min in the dark. Subsequently, cells were rinsed in phosphate buffer, dehydrated in increasing concentrations of ethanol, and embedded in Spurr resin. Ultrathin sections were cut on a Porter-Blum MT-2B ultramicrotome, stained with uranyl acetate and Reynold's lead solution, and examined using a Philips 300 transmission electron microscope (operating at 60 KV accelerating voltage).

*Oestrogen and progesterone receptors*

Cell pellets were prepared and stored in −80°C. A cytostolic extact was obtained from passage 5 on UACC-812 and passage 9 on UACC-893 and analysed as previously described (Kute *et al.*, 1980). Results are reported in femtomoles per mg of protein and results were defined as positive if there was >10 femtomole $mg^{-1}$ of protein and the affinity constant was $>5 \times 10^8$ $M^{-1}$. Positive and negative cytosolic material was analysed at the same time for quality control.

*Histochemistry*

Tissue cells were grown on coverslips, washing in PBS for 5 min, fixed in cold methanol for 5 min, and then in acetone for 5 s to assure permeabilisation. After 5 additional min in PBS, the cells were incubated with the desired antibody for 30 min, washed in PBS for 5 min and then incubated with fluorescinated goat anti mouse IgG (Cappel, Durham NC) for 30 min. Coverslips were then washed with PBS, mounted and observed with a Zeiss epifluorescent microscope. Antibodies were anticytokeratins KA1 and KA4 (Nagle *et al.*, 1986), anticytokeratin 10.11 (Chan *et al.*, 1986), anti-vimentin (Dako) and anti-HER-2 (Triton Biosciences). UACC was studied at passage 36 and UACC 893 was studied at passage 9.

*Cytogenetics*

Exponentially growing cultures were harvested for karyotypic analysis, slides prepared, and Q- or G-banding performed as previously described (Trent & Thompson, 1987). >50 cells per cell line were analysed, with results expressed according to ISCN recommendations (1985).

*Immunoblot analysis*

Plasma membrane preparations from UACC 812 and UACC 893 were prepared according to the method of Riordan and Ling (1979). Cell lines known to be negative (8226/S) and positive (8226/Dox40) for over expression of P-glycoprotein wre used as controls (Dalton *et al.*, 1986). Polyacrylamide gel electrophoresis was performed according to the method of Fairbanks *et al.* (1971) with slight modification (Delenham *et al.*, 1982). The procedure of Towbin *et al.* (1979) was used to transfer proteins from the gel to nitrocellulose paper which was then probed with the monoclonal antibody C-219 (Centocor). $^{125}$I-rabbit anti-mouse IgG (New England Nuclear Corp.) was used as a second antibody for detection by autoradiography.

In vitro *drug sensitivity testing*

A modified protein detection system was used to calculate cell survival following exposure to chemotherapeutic drugs (Bradford *et al.*, 1976). Single cell suspensions of the cell lines obtained in the exponential phase of cell growth were placed in the M-3 growth medium (Leibovitz, 1985) and plated at 30,000 cells/well in 96-well plates. Following a 5 day incubation with varying doses of drug, the media plus drug was aspirated from individual wells. Fifty µl of $H_2O$ was then added to each well, and after three freeze thaw cycles, 200 µl of Bio-Rad Protein Assay Reagent (Bio-Rad Laboratories) diluted 1:5 was added to each well. The absorbance of each well was read at 595 nm using a Dynatech MR600 microplate reader (Dynatech Laboratories). Controls included medium plus the highest drug concentration and cells with medium only. The percentage of survival was calculated by dividing the absorbance of drug treated cells by the absorbance of control cells.

*Isolation of DNA and Southern blotting*

High molecular weight genomic DNA was isolated by SDS-proteinase K lysis, organic extraction, and NaCl-ethanol precipitation (Maniatis *et al.*, 1982). The DNA was quantitated by the 4, 6-diamine 2-phenylindole dihydrochloride method (Kapuscinski & Skoczylas, 1977). Ten µg of DNA was digested with *Hind* III, electrophoreses in 0.8% agarose, and transferred to a Gene Screen membrane (Southern, 1975). Membranes were hybridised with the 440 bp Kpnl-Xbal fragment of HER-2, pKX044 (kindly provided by T. Yamamoto, University of Tokyo) labelled by the method of Feinberg and Vogelstein (1984).

### Results

*DNA flow cytometry*

Both neoplasms were aneuploid; the original breast tumour tissue and cell lines were in agreement; UACC-812 had a DNA index of 1.59, UACC-893 DNA index was 1.72.

*Establishment of cell lines*

The UACC-812 biopsy specimen yielded 1.8 × 10$^7$ viable cells and growth was obtained on all media (M19, 33 and 41) tested; the best growth was on medium 41 which was used to establish the cell line. However, by the 6th passage, the cells could be maintained on M13, 5% FBS or L15, 5% FBS. Aliquots were frozen down under liquid nitrogen in 2nd, 5th, 10th, 15th and 20th passages. There was minimal stromal contamination, and cells were first subcultured in 3 weeks.

The UACC-893 biopsy specimen yielded 2.1 × 10$^6$ viable cells. Initial tumour-like epithelial cells were evident in the initial outgrowth of all media tested (M15, 19, 41, 49, 50, 51, 52) but degenerated in all flasks except in one (M41) where a few colonies persisted and started to proliferate. Fibroblast-like cells started to overgrow the tumour colonies, but were eradicated by geneticin treatment. It required 4 months before these cells could be successfully subcultured. Cells were frozen down in the 3rd, 6th, 7th, 10th, 15th and 20th passages. By the 9th passage, the less complex medium M19 could readily be used for maintenance, but the cells still required pituitary extract for optimal growth. By the 13th subculture, M13 or L15, with 10% foetal bovine serum sufficed.

By light microscopy, both cell lines demonstrate an epithelioid morphology *in vitro* (including distinct nuclei and nucleoli and a cobblestone 3-dimensional growth) that is retained throughout numerous subcultures (Figure 2). Both UACC-812 and UACC-893 grow as slowly expanding colonies with defined boundaries. Cells at confluence grow predominantly 3-dimensionally as monolayers with multilayering. Occasionally, there was doming of cells. The cell

**Figure 2** Morphology: both UACC-812 and UACC-893 grow as slowly expanding colonies of epithelial-like cells. **a**, light microscopy and **b**, phase microscopy of cell line UACC-812. **c**, Light microscopy of cell line UACC-893. Final magnification 160 ×.

doubling time for UACC-812 is 100 h, while the UACC-893 cell line displays a doubling time of 120 h. Both cell lines have now been subcultured >50 times.

*Electron microscopy*

Ultrastructural analysis of both cell lines further documents the epithelioid nature of both cell lines (Figure 3). Electron microscopy performed at passage 6 for UACC-812 and passage 13 for UACC-893 demonstrated specialised junctions, including desmosomes as well as cytoplasmic filaments, multivesicular bodies, and prominent surface microvilli.

*Histochemistry*

UACC-812 (passage 36) and UACC-893 (passage 9) had identical reactivity on immunohistochemical analysis (Table III). Both were vimentin negative, diffusely positive for cytokeratin, and HER-2 positive.

*Oestrogen and progesterone receptors*

A portion of the biopsy sampled and cultured cells (from passages 5 and 9 of UACC-812 and passage 13 of UACC-893) were submitted for oestrogen and progesterone receptor analysis. Scatchard analysis performed on both patient biopsy samples demonstrated the primary tumours to be negative for ER and PR, a finding consistent with that observed for both tumour cell lines (data not shown).

*Cytogenetics*

Chromosome banding analysis was performed on the initial outgrowth on UACC-812 within 7 days of explanting and repeated at 3rd and 10th passage with similar findings. UACC-893 cytogenetics are from the original outgrowth within 7 days of explanting. Figures 4 and 5 document the unique karyotypic features of both tumours, confirming the independent origin of both lines. Both lines predominantly display cells with a near triploid modal chromosome number and numerous clonal structural chromosome abnormalities. The modal chromosome number of the UACC-893 cell line was 62, with a range of 51–65 chromosomes per cell. The karyotype (Figure 4) was characterised by numeric alterations (most notably chromosome loss) and the presence of seven unidentifiable marker chromosomes. Clonal structural alterations included: t(1;3)(p11;p11); t(1;7)(p13;q11); iso(6p); del(6)(q23); iso(7q);del(11)(p13); and t(11;?)(p14;?).

The karyotype of UACC-812 demonstrated a modal range of 58–64 and displayed numerous numeric and structural changes. As illustrated in Figure 5, the presence of multiple unidentifiable marker chromosomes, rings, and an abnormal chromosome 3 (~2.2 × the side of a normal 3) were observed. This latter chromosome was formed from the generation of an abnormally banding region on chromosome 3p. Clonal structural changes for UACC-812 included: del(2)(q24); t(2;?)(q37;?); HSR(3)(p21); del(7)(q22);t(7;?)(q36;?); iso(8q); del(9)(p13); del(11)(p13); t(21;?)(q22;?).

*HER-2/neu amplification*

Figure 6 shows a Southern blot of *Hind*III digested DNA from normal placenta and the UACC-812 and UACC-893 cell lines, and evidence for HER-2/neu amplification can be seen in DNAs from both tumour cell lines. The degree of amplification was estimated as ~15-fold for UACC-812 and ~20-fold for UACC-893 relative to placental DNA by Southern blotting of serial dilutions of tumour cell line DNA.

*P-glycoprotein*

Both cell lines were examined for the presence of P-glycoprotein using immunoblot analysis and the monoclonal antibody C-219. Neither UACC-812 nor UACC-892 produced P-glycoprotein at a detectable level using this assay (data not shown).

**Figure 3** Electron microscopy of UACC-812 **a** & **b** and UACC-893 **c** & **d**. Both cell lines demonstrated an ultrastructure profile consistent with breast epithelium including desmosomes (D); cytoplasmic filaments (CF); multivesicular bodies (MF); pseudo-inclusion (PI); and microvilli (V).

**Table III** Histochemical results

| | *Cytokeratins 5 and 14*[a] | *Cytokeratins 14, 15, 16 19*[b] | *Cytokeratins 8 and 18*[c] | *Vimentin* | *c-erbB-2* |
|---|---|---|---|---|---|
| UACC 812 | Negative | 4 + diffuse | 4 + diffuse | Negative | Positive |
| UACC 893 | Negative | 4 + diffuse | 4 + diffuse | Negative | Positive |

[a]Antibody KA1; [b]Antibody KA4; [c]Antibody 10.11.

**Figure 4** Giemsa-banded karyotype from UACC-893. A possible HSR can be seen on the second unidentifiable marker chromosome at the bottom of the karyotype.

*Drug sensitivity*

Figure 7 shows the cell survival curves for the two patient cell lines, compared to the well-known breast cancer cell line, MCF-7, when continuously exposed to the chemotherapeutic drug doxorubicin. Both patient cell lines were relatively resistant to this drug compared to the MCF-7 cell line.

### Discussion

Cell lines from breast tissue are among the most difficult to establish in tissue culture (Table I). We describe here two new breast carcinoma cell lines – UACC-812 and UACC-893 – both of which display amplification of the HER-2/neu gene. Both cell lines display morphology consistent with breast carcinoma, including ultrastructural features such as desmosomes and prominent surface microvilli. Both lines express cytokeratins, confirming their epithelial derivation. Also, both are hormone receptor negative, a feature held in common with the overwhelming majority of breast carcinoma cell lines established to date (Table I). Both lines carry amplified copies of the HER-2/neu gene and express the HER-2/neu protein. The frequent finding of HER-2/neu amplification in human breast cancers has recently been demonstrated (Slamon *et al.*, 1987) and amplification of this gene has been suggested to play an important role in clinical outcome.

Finally, despite recent reports of P-glycoprotein expression in clinical patient samples (Salmon *et al.*, 1989), the two lines we have established failed to significantly express this protein. Cell line UACC-893 was derived from a patient with primary Stage II breast cancer, whereas UACC-812 was obtained from a patient with recurrent metastatic disease, Stage II, grade IV, who had received extensive prior cytotoxic chemotherapy. It is possible that alternate mechanisms of multidrug resistance (Slovak *et al.*, 1988; Mirski *et al.*, 1987; Batist *et al.*, 1986) may be responsible for the relative drug resistance observed *in vitro* and the clinical drug resistance observed for the patient with Stage IV disease (UACC-812). Alternatively, it is equally possible that the long duration of cell culture required prior to cell passage and analysis for both lines eliminated cells differentially expressing P-glycoprotein.

The success rate of establishing cell lines from tumours in breast tissue has not progressed significantly since Lasfargues and Ozello established the first one over 30 years ago (1958). Only 13 additional cell lines have been added in spite of intensive effort by many investigators (Table I). The common features of all established cell lines including ours are: all are hyperdiploid; when determined by the authors, all had metas-

**Figure 5** Representative G-banded karyotype from UACC-812 showing 3pHSRand other clonal structural and numeric abnormalities. Ring chromosomes at the bottom of the karyotype are from other cells but were a clonal change in a small sideline population.

**Figure 6** Southern blot of *Hind*III cleaved DNA from UACC-812 and UACC-893 demonstrates amplification of the HER-2/neu gene relative to human placenta (HP). Ten μg of DNA was loaded in each lane and the blot was hybridised with the HER-2/neu probe pKX044.

**Figure 7** Cytotoxicity curves for the patient cell lines UACC-812 and UACC-893 compared to the breast cancer cell line MCF-7 when exposed to increasing doses of doxorubicin.

tasised *in vivo*; and the tumour tissue released viable tumour cells in relatively large numbers for culture.

In our laboratory, only these two out of about 100 primary breast specimens have resulted in established cell lines. However, more than 80% of the specimens yielded $<1 \times 10^6$ total viable cells (stromal and tumour cells). Most of these were in a milieu of dead and dying cells; the average viability of cell yields was about 20% with a range of $<1$% to 50%. Short-term cultures were obtained in approximately 80% of specimens. Typical adenocarcinoma-type islands of epithelial-like cells which were present in most instances, were useful for obtaining cytogenetic studies shortly after explantation, but invariably these died out within a few weeks. The specimens yielding cell lines were from fairly large tumours (UACC-812 had over $10^7$ viable tumour cells and UACC-893 had over $10^6$ viable tumour cells).

Three other cell lines (Table I) – VHB-1 (Vandewalle *et al.*, 1987), CAL 18A and CAL 18B (Gioanni *et al.*, 1985) – were derived from patients with a similar clinical history as our UACC-812. The patients refused surgical intervention for their original tumour and were treated by radiation and/or chemotherapy with clinical remission. However, after about 2 years, relapses occurred with carcinomas developing in the same breast or the other breast as well as metastasis to other sites. These tumour cells adapted readily to *in vitro* growth and cell lines were established with little difficulty.

Our experience in establishing the UACC-893 cell line duplicates the findings of Lasfargues *et al.* (1978) for BT-474 and Yamane *et al.* (1984) for YMB-1. Although tumour-like cells were evident on original explants, most of them died out and the cell lines were established from small clusters of tumour cells that persisted and finally started to proliferate. Four to 5 months elapsed before a successful subculture could be made.

Kraus *et al.* (1987) found four of 11 established breast cell lines to have HER-2/neu amplification. These included one of three cell lines derived from breast tissue (BT474) and three of eight from pleural fluid (SK-BR-3, MDA-MB-361 and MDA-MB-453). This ~33% rate correlates reasonably well with studies done in primary tissue (Slamon *et al.*, 1987; Berger *et al.*, 1988).

In conclusion, our experience describes the successful establishment of two breast carcinoma cell lines. Because of the potential importance of the HER-2/neu gene to the biology (and clinical course) of breast cancer, these lines may represent a useful model to investigate the pathogenesis of this disease. These cultures will be transmitted to the American Type Culture Collection for distribution to interested investigators.

Research supported in part by CA-41183 and CA-48491.

### References

BATIST, G., TULPULE, A., SINHA, B.K., KATKI, A.G., MYERS, C.E. & COWAN, K.H. (1986). Overexpression of a novel anionic glutathione transferase in multidrug resistant human breast cancer cells. *J. Biol. Chem.*, **261**, 15544.

BERGER, M.S., LOCHER, G.W., SAURER, S. & 4 others (1988). Correlation of c-*erb*B-2 gene amplification and protein expression in human breast carcinoma with nodal status and nuclear grading. *Cancer Res.*, **48**, 1238.

BRADFORD, M.M. (1976). A rapid and sensitive method for the quantitation of microgram quantities of protein utilizing the principal of protein-dye banding. *Anal. Biochem.*, **72**, 248.

CHAN, R., ROSSITO, P.V., EDWARDS, B.F. & CARDIFF, R.D. (1986). Presence of proteolytically processed keratins in the culture of MCF-7. *Cancer Res.*, **46**, 6353.

DALTON, W.S., DURIE, B.G.M., ALBERTS, D.S., GERLACH, J.H. & GROSS, A.E. (1986). Characterization of a new drug resistant human myeloma cell line which expresses P-glycoprotein. *Cancer Res.*, **45**, 5125.

DELENHAM, P.G., KARTNER, N., SIMINOVITCH, L., RIORDAN, J.R. & LING, V. (1982). DNA mediated transfer of multiple drug resistance and plasma membrane glycoprote in expression. *Mol. Cell Biol.*, **2**, 881.

DOBRYNIN, Y.V. (1963). Establishment and characteristics of cell strains from some epithelial tumors of human origin. *J. Natl Cancer Inst.*, **31**, 1173.

FAIRBANKS, G., STECK, T.C. & WALLACH, D.H.F. (1971). Electrophoretic analysis of the major polypeptides of the human erythrocyte membrane. *Biochem.*, **10**, 2606.

FEINBERG, A. & VOGELSTEIN, B. (1984). A technique for labeling DNA restriction fragments to a high specific activity. *Annal. Biochem.*, **137**, 66.

GIOANNI, J., COORDI, A., LALANNE, C. & 5 others (1985). Establishment, characterization, chemosensitivity and radiosensitivity of two different cell lines derived from a human breast cancer biopsy. *Cancer Res.*, **45**, 1246.

HACKETT, A.J., SMITH, H.S., SPRINGER, E.L. & 4 others (1977). Two syngeneic cell lines from human breast tissue. The aneuploid mammary epithelial (Hs578T) and the diploid myoepithelial (Hs578Bst) cell lines. *J. Natl Cancer Inst.*, **58**, 1795.

HALABAN, R. & ALFANO, F. (1984). Selective elimination of fibroblasts from cultures of normal human melanocytes. *In vitro*, **20**, 447.

HAYFLICK, L. (1973). Subculturing human diploid fibroblast cultures. In *Tissue Culture Methods and Applications*, Kruse, P.F. Jr & Patterson, M.K. (eds), pp. 220–223. Academic Press, New York.

INTERNATIONAL SYSTEM FOR HUMAN CYTOGENETIC NOMENCLATURE (ISCN) (1985). *Cytogene. & Cell Genet.*, **21**, 1.

KISCHER, C.W., LEIBOVITZ, A. & PINDUR, J. (1989). The use of a transport medium (L15M15) for bulk tissue storage and retention of viability. *Cytotech*, **2**, 181.

KAPUSCINSKI, J. & SKOCZYLAS, B. (1977). Simple and rapid fluorometric method for DNA micro assay. *Anal. Biochem.*, **83**, 252.

KRAUS, M.H., PROPESCU, N.C., AMSBAUGH, C. & KING, C.R. (1987). Overexpression of the EGF-related proto-oncogene erbB-2 in human tumor cell lines by different molecular mechanisms. *EMBO J.*, **6**, 605.

KUTE, T.E., HUSKE, M.S., SHORE, A. & RHYME, A.L. (1980). Improvements in steroid receptor assays including rapid computer analysis of data. *Anal. Biochem.*, **103**, 272.

LASFARGUES, E.Y. & OZELLO, L. (1958). Cultivation of human carcinomas. *J. Natl Cancer Inst.*, **21**, 1131.

LASFARGUES, E.Y., COUTINHO, W.G. & REDFIELD, E.S. (1978). Isolation of two human tumour epithelial cell lines from solid breast carcinomas. *J. Natl Cancer Inst.*, **61**, 967.

LEIBOVITZ, A., STINSON, J.C., MCCOMBS, W.B., MCCOY, C.E., MAZUR, K.C. & MABRY, N.D. (1976). Classification of human colorectal adenocarcinoma cell lines. *Cancer Res.*, **36**, 4562.

LEIBOVITZ, A. (1985). The establishment of cell lines from human solid tumors. In *Advances in Cell Culture*, Maramorosch, K. (ed.), p. 249. Academic Press, New York.

LEIBOVITZ, A., DALTON, W., MASSEY, K., VILLAR, H. & TRENT, J. (1988). Establishment and characterization of two new breast carcinoma cell lines from primary tumors. *Proc. Amer. Assoc. Cancer Res.*, **29**, 24.

MANIATIS, T., FRITSCH, E.F. & SAMBROOK, J. (1982). *Molecular Cloning*. Cold Spring Harbor, New York: Cold Spring Harbor Laboratory.

MACKLIS, J.D., SIDMAN, R.L. & DAVIS-SHINE, H. (1985). Cross-linked collagen surface for cell culture that is stable, uniform, and is optically superior to conventional surfaces. *In Vitro Cell Dev. Biol.*, **21**, 189.

MARTORELLI, B. Jr, PARSHLEY, M.S. & MOORE, J.G. (1969). Effects of chemotherapeutic agents on two lines of human breast carcinomas in tissue culture. *Surg. Gyn. Obst.*, **128**, 1001.

MINAFRA, S., MORELLO, V., GLORIOSO, F. & 5 others (1989). A new cell line (8701-BC) from primary ductal infiltrating carcinoma of human breast. *Br. J. Cancer*, **60**, 185.

MIRSKI, S.E.L., GERLACH, J.H. & COLE, S.P.C. (1987). Multidrug resistance in a human small cell lung cancer cell line selected in Adriamycin. *Cancer Res.*, **47**, 2594.

NAGLE, R.B., BOCKER, W., DAVIS, J.R. & 4 others (1986). Characterization of breast carcinomas by two monoclonal antibodies distinguishing myoepithelial from luminal epithelial cells. *J. Histochem. Cytochem.*, **34**, 869.

NORQUIST, R.E., ISHMAEL, D.R., LOVING, C.A., HYDER, D.M. & HOGE, A. (1975). The tissue culture and morphology of human breast tumor cell line BOT-2. *Cancer Res.*, **35**, 3100.

RIORDAN, J.R. & LING, V. (1979). Purification of P-glycoprote in from plasma membrane vesicles of Chinese hamster ovary cell mutants with reduced colchicine permeability. *J. Biol. Chem.*, **254**, 12701.

SALMON, S.E., GROGAN, T.M., MILLER, T.M., SCHEPER, R. & DALTON, W.S. (1989). Prediction of doxorubicin resistance *in vitro* in myeloma, lymphoma and breast cancer by P-glycoprotein staining. *J. Natl Cancer Inst.*, **81**, 696.

SLAMON, D.J., CLARK, G.M., WONG, S.G., LEVIN, W.J., ULLRICH, A. & MCGUIRE, W.L. (1987). Human breast cancer: correlation of relapse and survival with amplification of the HER-2/neu oncogene. *Science*, **325**, 177.

SLOVAK, M.L., HOELTGE, G.A., DALTON, W.S. & TRENT, J.M. (1988). Pharmacologic and biologic evidence for differing mechanisms of doxorubicin resistance in two human tumor cell lines. *Cancer Res.*, **48**, 2793.

SMITH, H.E., WOLMAN, S. & HACKETT, A.J. (1984). The biology of breast cancer at the cellular level. *Biochem. Biophys. Acta.*, **738**, 103.

SOUTHERN, E.M. (1975). Detection of specific sequences among DNA fragments separated by gel electrophoresis. *J. Mol. Biol.*, **98**, 503.

TOWBIN, H., STAEHELIN, T. & GORDON, J. (1979). Electrophoretic transfer of proteins from polyacrylamide gels to nicrocellulose sheets: procedure and some applications. *Proc. Natl Acad. Sci. USA*, **76**, 4350.

TRENT, J.M. & THOMPSON, F.H. (1987). Methods for chromosome banding of human and experimental tumors *in vitro*. In *Methods in Enzymology*, Academic Press (Molecular Genetics of Mammalian Cells), **151**, 267.

VANDEWALLE, B., D'HOOGHE, C., SAVARY, J.B. & 4 others (1987). Establishment and characterization of a new cell line (VHB-1) derived from a primary carcinoma. *J. Cancer Res. Clin. Oncol.*, **113**, 550.

YAMANE, M., NISHIKI, M., KATAOKA, T. & 7 others (1984). Establishment and characterization of a new cell line (YMB-1) derived from human breast carcinoma. *Hiroshima J. Med. Sci.*, **33**, 715.